# Efficacy of osimertinib against EGFRvIII+ glioblastoma

**DOI:** 10.18632/oncotarget.27599

**Published:** 2020-06-02

**Authors:** Gustavo Chagoya, Shawn G. Kwatra, Cory W. Nanni, Callie M. Roberts, Samantha M. Phillips, Sarah Nullmeyergh, Samuel P. Gilmore, Ivan Spasojevic, David L. Corcoran, Christopher C. Young, Karla V. Ballman, Rohan Ramakrishna, Darren A. Cross, James M. Markert, Michael Lim, Mark R. Gilbert, Glenn J. Lesser, Madan M. Kwatra

**Affiliations:** ^1^ Departments of Anesthesiology, Duke University Medical Center, Durham, NC, USA; ^2^ Genomic and Computational Biology, Duke University Medical Center, Durham, NC, USA; ^3^ Pharmacology and Cancer Biology, Duke University Medical Center, Durham, NC, USA; ^4^ Duke Cancer Institute, Duke University Medical Center, Durham, NC, USA; ^5^ Johns Hopkins Bloomberg School of Public Health, Johns Hopkins University School of Medicine, Baltimore, MD, USA; ^6^ Department of Dermatology, Johns Hopkins University School of Medicine, Baltimore, MD, USA; ^7^ Neurosurgery, Johns Hopkins University School of Medicine, Baltimore, MD, USA; ^8^ Department of Neurosurgery, The University of Alabama at Birmingham, Birmingham, AL, USA; ^9^ Tri-Institutional MD-PhD Program, Weill Cornell Medical College, The Rockefeller University, Memorial Sloan Kettering Cancer Institute, New York, NY, USA; ^10^ Department of Healthcare Policy and Research, Weill Cornell Medicine, New York, NY, USA; ^11^ Department of Surgery, Weill Cornell Medicine, New York, NY, USA; ^12^ IMED Oncology, Global Medical Affairs, AstraZeneca, Cambridge, UK; ^13^ Neuro-Oncology Branch, National Cancer Institute, National Institutes of Health, Bethesda, MD, USA; ^14^ Department of Internal Medicine, Section on Hematology and Oncology, Wake Forest School of Medicine, Winston-Salem, NC, USA

**Keywords:** EGFRvIII, tyrosine kinase, glioblastoma stem cells, osimertinib, xenografts

## Abstract

Epidermal Growth Factor Receptor variant III (EGFRvIII) is an active mutant form of EGFR that drives tumor growth in a subset of glioblastoma (GBM). It occurs in over 20% of GBMs, making it a promising receptor for small molecule targeted therapy. We hypothesize that poor penetration of the blood-brain barrier by previously tested EGFR-tyrosine kinase inhibitors (EGFR-TKIs) such as afateninb, erlotinib, gefitinib, and lapatinib played a role in their limited efficacy. The present study examined the effects of osimertinib (previously known as AZD9291) on EGFRvIII+ GBM models, both *in vitro* and *in vivo*. Therefore, a panel of six GBM stem cells (GSCs) expressing EGFRvIII+ was evaluated. The EGFRvIII+ GSC differed in the expression of EGFRvIII and other key genes. The GSC line D317, which expresses high levels of EGFRvIII and has robust tyrosine kinase activity, was selected for assessing osimertinib’s efficacy. Herein, we report that osimertinib inhibits the constitutive activity of EGFRvIII tyrosine kinase with high potency (<100 nM) while also inhibiting its downstream signaling. Further, osimertinib inhibited D317’s growth *in vitro* and in both heterotopic and orthotopic xenograft models. Additional preclinical studies are warranted to identify EGFRvIII+ GBM’s molecular signature most responsive to osimertinib.

## INTRODUCTION

Glioblastoma (GBM) is the most common and deadliest primary brain tumor. It carries a median survival of 16 months for newly-diagnosed patients whose treatment aligns with the current standard of care consisting of maximal safe resection followed by radiation and chemotherapy [1]. Although this treatment regimen improves overall survival, the benefit is modest and highlights the need for novel targeted therapies based on molecular classification. Accordingly, The Cancer Genome Atlas (TCGA) program recently performed the largest molecular characterization study of primary GBM tumors to date, using samples from over 500 patients [2, 3]. As a result of this study, it is now known that the Epidermal Growth Factor Receptor (EGFR) is extremely heterogeneous in GBM. At least four major molecular forms have been identified [2]: 1) wild-type EGFR with gene amplification; 2) EGFR with a large deletion in the extracellular domain (EGFRvIII); 3) EGFR with kinase domain duplication (EGFR-KDD); and 4) EGFR fused with SEPT-14 (EGFR-SEPT14). Also, several point mutations in the receptor’s extracellular and intracellular domains have been documented [2].

While a large body of evidence suggests that EGFR plays a significant role in GBM growth [4–6], previous efforts to target this receptor using EGFR-tyrosine kinase inhibitors (EGFR-TKIs) have been unsuccessful [7]. There are two central reasons why these agents may have been ineffective in treating GBM patients:1) all four FDA-approved EGFR-TKIs that have been tested so far in GBM patients (erlotinib [8, 9], gefitinib [8, 10], lapatinib [11], and afatinib [12]) do not cross the blood-brain barrier effectively; and 2) the extreme molecular and functional heterogeneity of EGFR, as mentioned above, could not be taken into account at the time these clinical trials were performed because this information was not yet understood [13–15]. Negative results in previous studies involving EGFR-TKIs in GBM patients can be in part explained by a failure to address the above two requirements.

As a result, our laboratory focuses on osimertinib (previously known as AZD9291) because it penetrates the blood-brain more effectively. Also, while it spares the wild-type EGFR it inhibits EGFRvIII tyrosine kinase with high potency [16]. The ability of osimertinib to inhibit EGFRvIII tyrosine kinase with high potency makes it an attractive candidate to target EGFRvIII, a driver of GBM growth that is present in over 20% of GBM patients [17]. Herein, we demonstrate that osimertinib inhibits the growth of EGFRvIII+ GBM expressing high EGFRvIII tyrosine kinase activity.

## RESULTS

### Brain penetration of osimertinib in athymic mice

**Figure 1:**
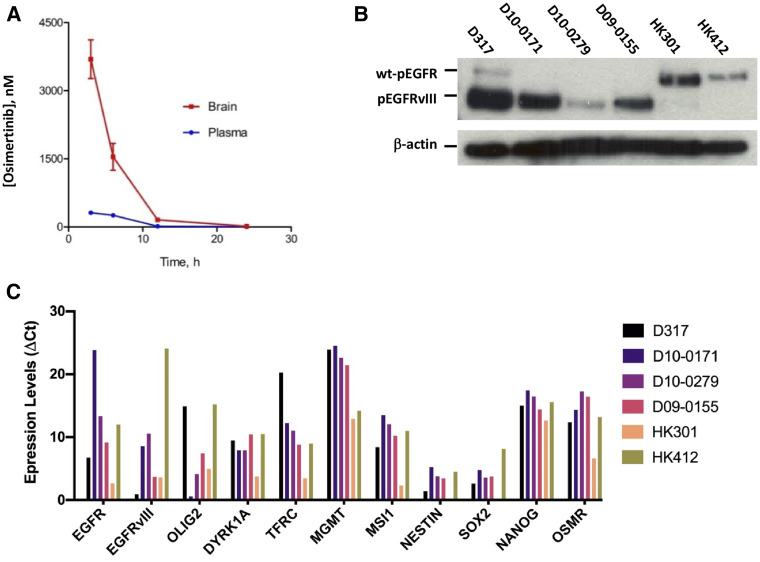
Pharmacokinetics of osimertinib and characterization of EGFRvIII+ GSCs. (**A**) Plasma and brain concentrations of osimertinib in athymic mice at different time points after a single oral dose of 25 mg/kg osimertinib. (**B**) Tyrosine kinase activity of wt-EGFR and EGFRvIII in our panel of six GSCs as determined by western blotting using an antibody specific for tyrosine phosphorylation at Y1068. (**C**) Expression of EGFR, EGFRvIII, and several other genes in our panel of six GSCs as determined by qPCR.

We first determined how well osimertinib penetrated the blood-brain barrier of athymic mice, a widely used preclinical model for cancer drug development [18]. As shown in Figure 1A, within three hours of a single 25 mg/kg oral dose, osimertinib accumulated in the brains of the mice to a concentration of 3,695 ± 425 nM, whereas its concentration in plasma was only 314 nM. Thus, in athymic mice, osimertinib has a brain to plasma ratio of >10. Osimertinib also rapidly penetrates the human brain [19] and is effective in lung cancer patients with brain metastases [20].

### Characterization of EGFRvIII+ glioblastoma stem cells (GSCs)

Because EGFRvIII has a deletion in its ligand-binding domain, its tyrosine kinase domain is constitutively active [21]. To detect the constitutive activity of EGFRvIII tyrosine kinase, we used western blotting with an antibody specific for EGFR tyrosine kinase phosphorylated at Y1068. These data are shown in Figure 1B. As Figure 1B shows, the constitutive tyrosine kinase activity of EGFRvIII in our panel of six EGFRvIII+ GSCs varied considerably. The highest tyrosine kinase activity is seen in D317 and D10-0171, followed by D09-0155 and D10-0279. Surprisingly, EGFRvIII’s constitutive tyrosine kinase activity is negligible in HK301 and HK412. These data indicate that EGFRvIII+ GBMs are heterogeneous in terms of EGFRvIII’s constitutive tyrosine kinase activity. To provide additional evidence of heterogeneity of EGFRvIII+ GBMs, we examined the expression of several key genes using qPCR. These data show that EGFRvIII+ GBMs differ in the expression of wt-EGFR and EGFRvIII as revealed by the Ct (threshold cycle) values; higher Ct values indicate lower expression. The expression of EGFRvIII was highest in D317 (Ct = 17.00) and lowest in HK412 (Ct = 36.72), explaining the differences in constitutive tyrosine kinase activities shown in Figure 1B. In addition, the EGFRvIII+ GBMs differed in the expression of several other genes notably OLIG2, a gene known to determine EGFR-TKI’s efficacy against EGFR expressing GBMs [22]. OLIG2 expression has been shown to negatively regulate EGFR expression [22] and our data reveals a similar relationship between OLIG2 and EGFR/EGFRvIII expression (Figure 1C).

### Efficacy of osimertinib against D317 which expresses high levels of EGFRvIII

**Figure 2:**
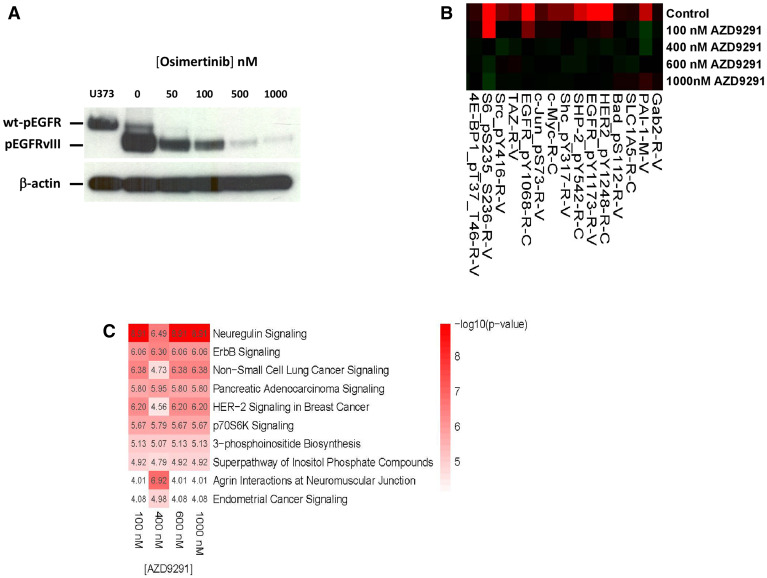
Effect of osimertinib on EGFRvIII tyrosine phosphorylation and downstream signaling. (**A**) Osimertinib blocks Y1068 phosphorylation of EGFRvIII, with an IC50 <100 nM. Lysate from EGF-stimulated U373 glioblastoma cells was used as a marker of wt-EGFR. (**B**) Lysates from control and osimertinib-treated D317 cells were analyzed by RPPA. Shown is a segment of the heatmap, which demonstrates inhibition of phosphorylation of EGFR Y1173 and several other proteins upon osimertinib treatment. (**C**) The RPPA data from control and osimertinib-treated D317 cells were analyzed by Ingenuity Pathway Analysis software. Shown are the top 10 pathways affected by the osimertinib treatment.

As shown in Figure 2A, osimertinib potently inhibits the EGFRvIII’s tyrosine phosphorylation at Y1068 with an IC_50_ of around 50 nM. This value is similar to that reported for EGFRvIII recombinantly expressed in HEK293 cells [16]. We also loaded a sample of U373 GBM cells treated with EGF to visualize the mobility of wild-type EGFR with respect to the mobility of EGFRvIII on the western blot (Figure 2A).

To further characterize osimertinib’s inhibition of EGFRvIII tyrosine kinase in D317 GSCs, we subjected control and osimertinib-treated D317 cells to proteomic analyses by RPPA. We found that treatment of GSC D317 with osimertinib inhibited the phosphorylation of several proteins that were phosphorylated in the control or untreated GSC D317 (Figure 2B and Supplementary Table 1). These included EGFR itself and several other phosphoproteins such as Src and S6 kinase. In the case of EGFR, phosphorylation of the receptor at both Y1068 and Y1173 (rows 98 and 99, Supplementary Table 1) is blocked by osimertinib; the levels of total EGFR (row 97, Supplementary Table 1) are not affected by osimertinib. These data establish that osimertinib’s blockade of EGFRvIII tyrosine kinase leads to a blockade of EGFRvIII’s intracellular signaling. Ingenuity Pathway Analysis of RPPA data from control and osimertinib-treated GSC D317 revealed a blockade of several signaling pathways, including neuregulin, ErbB, HER2 signaling in breast cancer, and p70S6K signaling (Figure 2C). The complete RPPA data is included in the supplementary section.

### Effect of osimertinib on the growth of D317 GSC *in vitro* and in xenograft models

**Figure 3:**
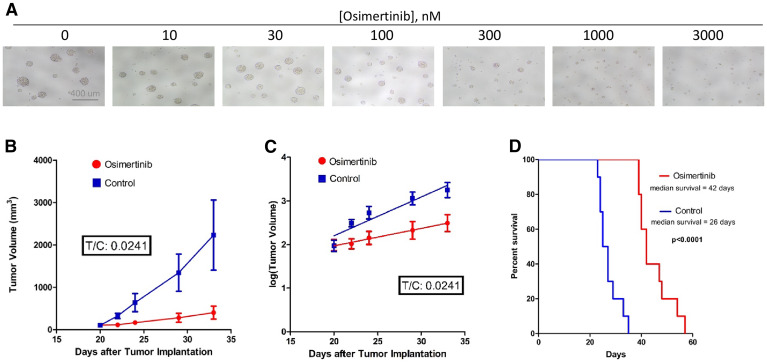
Osimertinib inhibits EGFRvIII+ GBM growth in cell culture as well as in xenograft models. (**A**) Osimertinib inhibits neurosphere formation and growth of EGFRvIII+ GSC D317. Freshly dissociated GSC D317 at a density of 2000-5000 cells/well in 200 ul media were plated in 96-well plates, and the cells were treated with different concentrations of osimertinib. After 5–7 days of treatment, cells were visualized under the microscope and photographed. (**B**) Tumor volume vs. time plot in control and treated mice. Athymic mice (*n* = 20) were injected with100-300K D317 cells subcutaneously, and treatment started once the tumors reached a size of 100–200 mm3 in diameter. Control animals (*n* = 10) received the vehicle, 0.5% HPMC (hydroxypropyl methyl cellulose), and treated animals (*n* = 10) received 25 mg/kg osimertinib, given by oral gavage once a day. Animals were sacrificed once tumor size reached 2000 mm3 in diameter. (**C**) Analysis of the tumor growth data shown in 3B using the rate-based T/C method. A rate-based T/C value below 0.4 indicates the treatment had a significant effect on tumor growth. (**D**) A group of athymic mice (*n* = 18) were injected with D317 cells intracranially. Treatment started 7 days after implantation. Control animals (*n* = 9) were treated with vehicle (0.5% HPC), while treated animals (*n* = 9) were given 25 mg/kg osimertinib by oral gavage twice a day. Animals were sacrificed when neurological symptoms appeared, including signs of motor disturbances and/or imbalance, decreased food intake, and/or signs of lethargy.

Figure 3A demonstrates that osimertinib inhibited the growth of D317 cells *in vitro*. Quantification of osimertinib’s ability to inhibit the growth of D317 GSCs using the WST-1 cell proliferation assay provided an IC_50_ of 476 ± 163 nM. These data show that osimertinib inhibits the growth of EGFRvIII+ GSCs at concentrations attainable in the brain (Figure 1A).

We next determined the ability of osimertinib to inhibit the growth of D317 GSCs using both heterotopic and orthotopic xenograft models. Figure 3B shows a plot of tumor volume versus days after subcutaneous injection of GSC D317 in control and osimertinib-treated mice. Tumor growth in the osimertinib-treated group was notably slower (Figure 3B). Analysis of these data using the rate-based T/C method (Figure 3C), which measures the rate of growth of tumor formation in control (C) and treated (T) animals, reveals a T/C of 0.0241. This is significant because in this analysis, a T/C <0.4 is considered to be significant growth inhibition [23].

The efficacy of osimertinib against tumors formed with GSC D317 in an orthotopic xenograft model is shown in Figure 3D. The data show that osimertinib was effective in slowing the growth of intracranial tumors. The median survival of untreated mice was 26 days, which increased to 42 days (p<0.0001) in osimertinib-treated mice.

To determine whether osimertinib would be effective against another EGFRvIII+ GBM, we selected D10-0171 GSCs (Figure 1C). EGFRvIII tyrosine kinase in D10-0171, like that in D317, is inhibited by osimertinib with high potency (IC_50_ <100 nM) (data not shown). Further, osimertinib inhibited the growth of D10-0171 in a subcutaneous model but the effect was modest (T/C of 0.1669).

## DISCUSSION

The present study confirms previous reports that osimertinib penetrates the blood-brain barrier effectively. Evaluation of a panel of six EGFRvIII+ GBMs revealed heterogeneity on the expression of EGFRvIII and in the extent of EGFRvIII’s tyrosine kinase activity. Evaluation of osimertinib’s efficacy against EGFRvIII+ GBMs with high expression of EGFRvIII and a robust EGFRvIII tyrosine kinase activity revealed that osimertinib inhibits the growth of these tumors effectively. Whether osimertinib will be effective against EGFRvIII+ GBMs with a lower expression of EGFRvIII and low EGFRvIII tyrosine kinase activity remains to be established.

The *in vitro* and *in vivo* data presented here demonstrate the ability of osimertinib to inhibit the growth of EGFRvIII+ GBMs with high EGFRvIII tyrosine kinase activity. This ability of osimertinib is based on several key properties. First, it penetrates the blood-brain barrier very well (Figure 1A). While our studies were on mice, osimertinib has been shown elsewhere to penetrate the human brain as well [19, 24–27]. Second, osimertinib is an irreversible inhibitor of EGFR tyrosine kinase, so its inhibition of EGFRvIII signaling is long-lasting. Third, osimertinib inhibits multiple intracellular pathways involved in cancer growth in EGFRvIII+ GBM (Figure 2C). These data suggest that osimertinib may be a better candidate than the previously tested EGFR-TKIs for GBM patients.

An important observation made in this study is that EGFRvIII+ GBMs are heterogeneous in terms of expression of EGFRvIII, the extent of EGFRvIII’s tyrosine kinase activity, and in the expression of several other key genes (Figures 1B and 1C). To our knowledge, previous clinical trials did not take into account the heterogeneity of EGFRvIII+ GBMs. For example, the failed EGFRvIII-vaccine trial included all EGFRvIII+ GBM patients [28]. We propose that future clinical trials involving EGFRvIII+ GBM patients should have the patients segregated into at least three groups: 1) a group with high constitutive activity of EGFRvIII’s tyrosine kinase, 2) a group with low constitutive activity of EGFRvIII’s tyrosine kinase, and 3) a group with negligible constitutive activity of EGFRvIII’s tyrosine kinase activity. The suggestion of having EGFRvIII+ GBM patients to be divided into three groups is based on our data shown in Figure 1B.

In summary, our study establishes that osimertinib penetrates the blood-brain barrier effectively and inhibits the growth of EGFRvIII+ GBMs with higher EGFRvIII tyrosine kinase activity. Additional preclinical studies are needed to define the molecular signatures of EGFRvIII+ GBMs that may make these tumors sensitive to osimertinib.

## MATERIALS AND METHODS

### Pharmacokinetic analysis of osimertinib in plasma and brain of athymic mice given a single oral dose

All animal protocols used in this study were approved and performed in accordance with guidelines set forth by the Institutional Animal Care & Use Committee (IACUC) under protocol number A134-17-05. Athymic nu/nu female mice aged 10 to 12 weeks and weighing 20–28 g were obtained from the breeding core at Duke University Medical Center. A group of sixteen mice was divided into two groups of eight mice each. Drug or vehicle was administered by oral gavage. The control animals were given 100 μl of 0.5% hydroxypropyl methylcellulose (0.5% HPMC), and osimertinib-treated animals were given 100 μl of 6.25 mg/mL osimertinib formulated in 0.5% HPMC. This corresponds to a 25 mg/kg osimertinib dose, using 25 g as the average mouse weight. Two mice were sacrificed from each group at 3, 6, 12, and 24 h after vehicle/drug administration. After sacrificing through CO_2_ euthanasia, the mice were decapitated and 100 μL of blood from each mouse was collected into EDTA-coated tubes. Additionally, brain samples from each animal were also collected. The blood samples were spun down at 10,000 rpm for 10 min, and the supernatant (plasma) was collected. Brain tissue and plasma samples were flash-frozen in liquid nitrogen and stored at –80° C until analyses for osimertinib levels by the PK/PD Core facility at Duke using LC/MS were performed.

### Isolation of GSCs from EGFRvIII+ GBMs

Two EGFRvIII+ GSCs (HK301and HK412) were generously provided by Dr. Harley Kornblum at the University of California at Los Angeles (UCLA: Los Angeles, CA). Four EGFRvIII+ GSCs (D317, D10-0171, D09-0155, and D10-0279) were isolated from EGFRvIII-expressing patient-derived xenografts (PDXs) developed at the Duke Brain Tumor Center and characterized [29]. To isolate GSCs, PDGXs were grown subcutaneously in nude mice. They were removed and placed into PBS with 10 μg/mL ciprofloxacin, minced, and digested with Liberase enzyme at 100 μg/mL for 5–10 min at 37° C. The cell suspension was filtered through a 70-micron sieve. Viable cells were separated from dead cells and tissue debris with Ficoll Paque Plus (GE Healthcare; Chicago, IL). The viable cells were extracted and washed with stem cell media (StemPro NSC media from Gibco Cat # A1050901; Life Technologies, Carlsband, CA) containing 20 ng/mL EGF and 20 ng/mL bFGF. Isolated stem cells were resuspended in fresh StemPro media and counted. Stem cells were plated at a density of 1 × 10^5^ cells/mL in stem cell media, and neurospheres formed within 24 to 48 h. Isolation of GSCs from PDGX has been reported previously [30, 31].

To passage GSCs, neurospheres were centrifuged at 200 g, washed with PBS, and resuspended in 2 mL of Accutase (Gibco Life Technologies, #A11105-01). After 10–15 min incubation at room temperature, 10 mL of StemPro media was added, and the suspension was gently mixed by pipetting. The resulting single-cell suspension was centrifuged, and the cell pellet was taken in 10 mL of fresh StemPro media, and cells were counted using a Countess II automated cell counter (Life Technologies). New 75 mm flasks were made using 2–4 × 10^6^ cells. Dissociated cells were plated in 96-well plates (2,000 to 5,000 cells/well) for cell viability studies.

For studying the effect of osimertinib on tyrosine kinase activity and for studying the effect of osimertinib on intracellular signaling by reverse-phase protein array (RPPA), about 800,000 cells were plated in each well of a six-well plate. For isolating RNA and for proteomic analysis, 3–5 million cells were centrifuged and washed twice with PBS, then flash-frozen in liquid nitrogen and stored at –80° C until processed.

### Characterization of EGFRvIII+ GSCs using qPCR

Using Taqman Gene Expression Assays, each GSC line was characterized for the expression of EGFR, EGFRvIII, DYRK1, OSMR, and MGMT as well as several stem cell markers, including the following: TFRC (transferrin receptor), SOX2, CD133, NESTIN, NANOG, MSI-1, and OLIG2. Briefly, total RNA was isolated from dissociated GSCs using the RNeasy^®^ Plus Mini Kit (Qiagen, Cat #74134; Hilden, Germany). cDNA was prepared using the Superscript^®^ VILO™ cDNA Synthesis Kit from Invitrogen (Cat #11754; Carlsbad, CA). To rule out the presence of genomic DNA, cDNAs were prepared both with and without reverse transcriptase (RT). The expression of various genes was determined using the Taqman^®^ Gene Expression Assays from Applied Biosystems/Life Technologies division of ThermoFisher Scientific. The following assays, with their catalog numbers in parentheses, were used: GAPDH (Hs99999905), EGFR (Hs01076090_m1), EGFRvIII (custom synthesized, Cat #4441117, Assay ID: AJPACOW), DYRK1 (Hs00176369_m1), OSMR (Hs00384276_m1), MGMT (Hs01037698_m1), TFRC (Hs00951083_m1), SOX2 (Hs01053049_s1), CD133 (Hs01009257_m1), NESTIN (Hs04187831_g1), NANOG (Hs04399610_g1), MSI-1 (Hs01045894_m1), and OLIG2 (Hs00377820_m1). Each transcript was tested under three conditions: NTC (no target control); - RT (cDNA prepared without RT); and + RT (cDNA prepared with RT). All reactions were performed in triplicate and under similar conditions. Ct values were measured using the StepOne instrument from Applied Biosystems (Foster City, CA). The Ct value of each gene was normalized by subtracting the Ct value of GAPDH, giving ΔCt values. These values are plotted as a bar graph (Figure 1C). Note: higher Ct values indicate lower expression of the transcript.

### Effect of osimertinib on tyrosine phosphorylation of EGFRvIII and its downstream signaling in GSC D317

Approximately 800,000 freshly dissociated GSC D317 cells in StemPro media were incubated with different concentrations of osimertinib in a 37° C, 5% CO_2_ incubator for six hours. Control and treated cells were centrifuged and the cell pellet was washed twice with ice-cold PBS. The washed cell pellets were taken in 100 μL of RPPA lysis buffer and protein concentration was measured using Bradford reagent. Tyrosine phosphorylation of EGFR/EGFRvIII was visualized by western blotting as described previously [29]. Samples for RPPA were prepared according to the instructions provided by the RPPA core facility at M. D. Anderson (Houston, TX) as described previously [29]. The RPPA data were analyzed by Ingenuity Pathway Analysis (IPA) suite. For each protein quantified with RPPA, we calculated the fold-change between the osimertinib samples and the control sample. Within each osimertinib concentration, we identified the proteins that showed at least a 50% change in their abundance relative to control. These proteins were then used in IPA (accessed 4/2/2018) to identify canonical pathways that are enriched for these genes.

### Effect of osimertinib on the growth of EGFRvIII+ GSC D317 neurospheres *in vitro* and subcutaneous and intracranial xenograft models

Freshly dissociated D317 GSCs were plated (5,000 cells/well) in a 96-well plate. The cells were treated with different concentrations of osimertinib and incubated for 5–7 days. The effect of osimertinib on D317 neurospheres was visualized using a microscope equipped with a camera. The ability of osimertinib to inhibit the growth of D317 GSCs was quantitated using the WST-1 Cell Proliferation Assay System (cat#MK400) from Takara Bio USA (Mountainview, CA) as per manufacturer’s protocol.

To study the effect of osimertinib D317 in a subcutaneous model, athymic mice (*n* = 20) were subcutaneously injected with freshly dissociated GSCs in 100 μl of StemPro media and Matrigel (1:1). Tumor growth was monitored visually 2-3 times per week. After the tumor had shown established growth (at the size of 50–200 mm^3^), mice were randomly divided into control (*n* = 10) and osimertinib-treated (*n* = 10) groups. Control animals received 100 μl of the vehicle in 0.5% HPMC and treated animals received 100 μl of 6.25 mg/mL osimertinib in 0.5% HPMC daily, by oral gavage. This resulted in a dose of 25 mg/kg using 25 g as the average weight of mice. Tumor volumes were measured twice a week, using hand-held Vernier calipers (Scientific Products; McGraw, IL). Tumor volume, V, was calculated with the formula V = [(width)^2^ x (length)]/2 ([mm^3^]). Animals were sacrificed when their tumors reached the maximum allowed by IACUC’s tumor size limit. The data were analyzed using a Rate-Based Treatment/Control (T/C) test, which is preferable to the Traditional T/C test because it uses many data points from different days and is less dependent on the length of the study [23, 32]. To do this analysis, we transformed the y values to log (y) due to the assumption that tumors grow exponentially (Figure 3B). Linear regression was then applied, and the mean slopes were used in the following formula: Rate-based *T* /*C* = 10^(mT^ ^-μC^ ^) × # days^ where μ_T_ is the slope of the treated data and μ_C_ is the slope of the control data. A T/C value below 0.4 is considered significant [23].

### To study the effect of osimertinib on the survival of mice bearing intracranially implanted GSCs D317

Freshly dissociated GSC cells in 5 μL StemPro media were implanted in the brain of athymic mice (*n* = 18) as follows: one hour before implantation, immunocompromised mice were given 100 μL of 0.5 mg/mL meloxicam subcutaneously to control pain and inflammation. For intracranial implantation, a mouse was anesthetized with isoflurane and placed into a mouse stereotactic frame with its head secured, receiving constant isoflurane. Betadine was applied on top of the head and a 1 cm sagittal incision was made along the scalp. A cleaning Q-tip was used to help expose the bregma. A Hamilton syringe with a 23G Luer needle was inserted, 2 mm laterally of the bregma, to a depth of 5.0 mm into the skull, then pulled back up 4.0 mm before injecting. The cells were administered over 2.5 minutes. Bone wax was applied to the skull generously, and the skin was stapled. The mouse cage was placed onto a heating pad and the animals were observed until they ambulated. The cage was then returned to its rack. A second dose of meloxicam was delivered subcutaneously 24 hours post-surgery. Treatment with vehicle and osimertinib was started 7 days after tumor cell implantation, with nine mice in each treatment group. Animals were given 25 mg/kg osimertinib by oral gavage twice a day. Animals were observed daily, weighed every 3–4 days, and sacrificed upon developing neurologic symptoms, including being severely hunched, being lethargic, and/or lacking movement. The survival data were analyzed using GraphPad (GraphPad Software, Inc.; San Diego, CA).

## SUPPLEMENTARY MATERIALS




