# Supplementary material for: Efficacy of osimertinib against EGFRvIII+ glioblastoma
Source: Oncotarget. 2020 Jun 2;11(22):2074–82. doi: 10.18632/oncotarget.27599 (PMC7275784; doi:10.18632/oncotarget.27599)
Supplement: Supplementary file 1 [file oncotarget-11-2074-s001.pdf]

Efficacy of osimertinib against EGFRvIII+ glioblastoma

SUPPLEMENTARY MATERIALS

Supplementary Table 1: Effect of various concentrations of osimertinib on protein expression (Normal Linear) in D317 GBM stem cells. See Supplementary Table 1
