# Supplementary material for: Efficacy of osimertinib against EGFRvIII+ glioblastoma
Source: Oncotarget. 2020 Jun 2;11(22):2074–82. doi: 10.18632/oncotarget.27599 (PMC7275784; doi:10.18632/oncotarget.27599)
Supplement: Supplementary file 2 [file oncotarget-11-2074-s002.docx]

Supplementary Table 1. Effect of Various Concentrations of Osimertinib on Protein Expression (Normal Linear) in D317 GBM Stem Cells

|  | **Antibody Name** | **Gene Name** | **Validation Status** | **[Osimertinib], nM** | | | | |
| --- | --- | --- | --- | --- | --- | --- | --- | --- |
|  |  |  |  | 0 | 100 | 400 | 600 | 1000 |
| 1 | 14-3-3-beta | YWHAB | V | 0.98566376 | 1.02742511 | 1.14150048 | 1 | 1.01719145 |
| 2 | 14-3-3-epsilon | YWHAE | C | 1 | 0.99365695 | 1.02758067 | 0.97803529 | 1.01170386 |
| 3 | 14-3-3-zeta | YWHAZ | V | 1.01761426 | 1.06332314 | 0.93040138 | 1 | 0.97119768 |
| 4 | 4E-BP1 | EIF4EBP1 | V | 1.02803102 | 0.98071378 | 1.08766162 | 0.9660757 | 0.9961674 |
| 5 | 4E-BP1_pS65 | EIF4EBP1 | V | 1.06447096 | 0.98969593 | 1.00458521 | 0.93024669 | 0.9961674 |
| 6 | 4E-BP1_pT37_T46 | EIF4EBP1 | V | 1.12974164 | 1.05836112 | 0.97362786 | 0.98237088 | 0.9961674 |
| 7 | 53BP1 | TP53BP1 | V | 0.99915639 | 0.93239507 | 1.21540766 | 1 | 1.00026775 |
| 8 | A-Raf | ARAF | V | 1.00708157 | 0.95938628 | 0.87447146 | 1 | 0.9640145 |
| 9 | ACC1 | ACACA | C | 1 | 0.97646497 | 0.61245053 | 1.04519686 | 1.06298497 |
| 10 | ACC_pS79 | ACACA | V | 1 | 0.85929101 | 0.85306601 | 1.00154839 | 1.04734534 |
| 11 | ACVRL1 | ACVRL1 | C | 0.94975341 | 0.97594466 | 0.94199391 | 1 | 0.9978774 |
| 12 | ADAR1 | ADAR | V | 0.92939501 | 0.96376381 | 0.96453129 | 1.02274241 | 0.9961674 |
| 13 | Akt | AKT1 | V | 1 | 0.98592362 | 1.40322819 | 0.97926398 | 1.10648062 |
| 14 | Akt_pS473 | AKT1 | V | 1.41678977 | 0.99263288 | 1.17859637 | 0.96584923 | 0.9961674 |
| 15 | Akt_pT308 | AKT1 | V | 1 | 1.001507 | 1.27546461 | 0.97475756 | 1.0198371 |
| 16 | AMPKa | PRKAA1 | C | 0.9999256 | 0.97652173 | 0.92806012 | 1 | 1.09487115 |
| 17 | AMPKa_pT172 | PRKAA1 | C | 1 | 0.95697812 | 0.86785087 | 0.95989132 | 1.03868242 |
| 18 | Annexin-I | ANXA1 | V | 1.0911132 | 0.94597087 | 0.76230292 | 1.05830224 | 0.9961674 |
| 19 | Annexin-VII | ANXA7 | V | 0.93870227 | 0.93954738 | 0.86476433 | 1.0527408 | 1.01756316 |
| 20 | AR | AR | V | 0.98386686 | 0.95996728 | 0.9697469 | 1 | 0.99742319 |
| 21 | ARHI | DIRAS3 | C | 0.99551313 | 1.09404582 | 1.18919735 | 1.08342441 | 0.9961674 |
| 22 | ARID1A | ARID1A | C | 1.07278843 | 0.99731229 | 0.80574398 | 1.08520276 | 0.9961674 |
| 23 | Atg3 | ATG3 | V | 1.10537323 | 1.02042412 | 0.89007808 | 0.9844369 | 0.9961674 |
| 24 | Atg7 | ATG7 | V | 0.96752114 | 1.04794987 | 0.9231501 | 1.01662404 | 0.9961674 |
| 25 | ATM | ATM | V | 1.00001716 | 0.98101102 | 0.86476433 | 1.03158862 | 0.97942689 |
| 26 | ATM_pS1981 | ATM | V | 1.00881823 | 1.05038985 | 1.16749318 | 0.97678131 | 0.9961674 |
| 27 | ATP5A | ATP5A | C | 1 | 0.83313341 | 1.10472282 | 0.7179607 | 1.01641074 |
| 28 | ATR_pS428 | ATR | C | 1.02521603 | 1.04294919 | 1.09650066 | 0.98444531 | 0.9961674 |
| 29 | Aurora-B | AIM1 | V | 0.90160177 | 0.95891208 | 0.97518117 | 1 | 1.01643541 |
| 30 | Axl | AXL | V | 1 | 1.00438809 | 1.13095288 | 0.99749263 | 1.08910605 |
| 31 | b-Actin | ACTB | C | 0.98708908 | 0.92816129 | 1.99650475 | 1 | 1.08142729 |
| 32 | b-Catenin | CTNNB1 | V | 0.97878928 | 0.94282792 | 1.55357873 | 1 | 1.10353415 |
| 33 | b-Catenin_pT41_S45 | CTNNB1 | V | 0.99504548 | 1.00750063 | 1.15027854 | 1 | 1.03442507 |
| 34 | B-Raf | BRAF | V | 1 | 1.00525754 | 1.11011687 | 0.9888648 | 1.12531004 |
| 35 | B-Raf_pS445 | BRAF | V | 0.96540726 | 0.95869611 | 1.03973663 | 1 | 1.02022989 |
| 36 | B7-H3 | CD276 | C | 1.02326986 | 0.88441252 | 0.75506553 | 1 | 1.04515235 |
| 37 | B7-H4 | VTCN1 | C | 0.91076118 | 1.10987414 | 0.82873803 | 1.13668659 | 1.07804588 |
| 38 | Bad_pS112 | BAD | V | 1.05550896 | 0.94776077 | 0.92328509 | 0.91268204 | 0.9961674 |
| 39 | Bak | BAK1 | C | 1 | 0.97310558 | 0.89075183 | 0.92444257 | 0.99726163 |
| 40 | BAP1 | BAP1 | V | 1.0472757 | 1.00847854 | 0.86476433 | 1.00765546 | 0.99087908 |
| 41 | Bax | BAX | V | 0.98989099 | 0.96057747 | 1.27156941 | 1 | 1.10329268 |
| 42 | Bcl-xL | BCL2L1 | V | 1 | 0.92734857 | 1.2628059 | 0.99809236 | 1.1435226 |
| 43 | Bcl2 | BCL2 | V | 0.97857253 | 1.04320396 | 0.88813511 | 1 | 1.00319329 |
| 44 | Bcl2A1 | BCL2A1 | V | 0.96271417 | 0.99764789 | 1.06587879 | 1 | 1.00427721 |
| 45 | Beclin | BECN1 | C | 1 | 1.04141389 | 1.01506281 | 0.95325044 | 1.05104961 |
| 46 | Bid | BID | C | 1 | 1.01219918 | 1.20263583 | 0.99625601 | 1.14952802 |
| 47 | Bim | BCL2L11 | V | 0.9250023 | 0.92983017 | 0.93917978 | 1 | 1.02952793 |
| 48 | BRD4 | BRD4 | V | 1.01770926 | 0.91267306 | 0.79979391 | 1 | 1.01010752 |
| 49 | c-Abl | ABL1 | V | 1 | 1.019313 | 1.21658493 | 0.97578604 | 1.01186332 |
| 50 | c-Jun_pS73 | JUN | V | 1.23043535 | 1.05648153 | 0.97560029 | 1 | 0.99150504 |
| 51 | c-Kit | KIT | V | 0.96045363 | 1.0358503 | 1.06810455 | 1 | 1.02688383 |
| 52 | c-Met | MET | Q | 0.96862108 | 1.01476175 | 1.05570299 | 1 | 1.04491599 |
| 53 | c-Met_pY1234_Y1235 | MET | V | 0.93392729 | 0.95284301 | 1.0098764 | 1 | 1.03162493 |
| 54 | c-Myc | MYC | C | 1.39081151 | 1.06723589 | 0.97030393 | 1 | 0.98603373 |
| 55 | C-Raf | RAF1 | C | 0.99411532 | 0.96024703 | 0.88388895 | 1 | 1.00336929 |
| 56 | C-Raf_pS338 | RAF1 | V | 0.99024491 | 1.03752884 | 1.1861907 | 1.02561275 | 0.9961674 |
| 57 | Caspase-3 | CASP3 | C | 0.91822314 | 0.96645923 | 1.06787438 | 1 | 1.029349 |
| 58 | Caspase-7-cleaved | CASP7 | C | 0.90034597 | 0.96464662 | 1.24428634 | 1 | 1.04604312 |
| 59 | Caspase-8 | CASP8 | Q | 0.94692061 | 0.95268374 | 0.96620085 | 1.00712387 | 0.9961674 |
| 60 | Caveolin-1 | CAV1 | V | 1 | 0.95300066 | 0.77864531 | 1.04965565 | 1.03881343 |
| 61 | CD171 | L1CAM | V | 1 | 1.05607277 | 0.83071511 | 1.06760349 | 1.09745518 |
| 62 | CD26 | DPP4 | V | 1 | 0.9915704 | 1.2556192 | 0.9770815 | 1.09001057 |
| 63 | CD29 | CD29 | V | 0.92286714 | 0.93479528 | 0.94485347 | 1.00883039 | 0.9961674 |
| 64 | CD31 | PECAM1 | V | 1 | 1.01973587 | 0.8431231 | 1.0596394 | 1.01483818 |
| 65 | CD44 | CD44 | C | 1.06746858 | 0.95145463 | 1.16625448 | 0.95662344 | 0.9961674 |
| 66 | CD49b | ITGA2 | V | 1 | 1.05846926 | 1.17309288 | 1.02712943 | 0.98905135 |
| 67 | cdc25C | CDC25C | V | 1 | 1.0386442 | 0.99712229 | 1.004383 | 0.97740397 |
| 68 | CDK1 | CDK1 | C | 1.04336532 | 0.96563659 | 0.8939831 | 1 | 0.93076433 |
| 69 | Chk1 | CHEK1 | C | 1.26114439 | 1.10987414 | 0.70848671 | 1.05738099 | 0.83692636 |
| 70 | Chk1_pS296 | CHEK1 | V | 0.92474611 | 1.0529882 | 0.98575896 | 1.01722925 | 0.9961674 |
| 71 | Chk1_pS345 | CHEK1 | C | 0.99416232 | 0.98842946 | 1.28363191 | 1 | 1.04746197 |
| 72 | Chk2 | CHEK2 | V | 1.00799465 | 1.04554177 | 0.9567968 | 0.95762547 | 0.9961674 |
| 73 | Chk2_pT68 | CHEK2 | C | 1 | 1.01461078 | 0.98432311 | 0.98284787 | 1.01732978 |
| 74 | Claudin-7 | CLDN7 | V | 1 | 1.06717756 | 1.00298617 | 1.00833467 | 0.98609461 |
| 75 | COG3 | COG3 | V | 0.99968591 | 1.00979244 | 1.03834455 | 1 | 1.02108849 |
| 76 | Collagen-VI | COL6A1 | V | 0.99317953 | 1.05868316 | 0.99118831 | 1.01702486 | 0.9961674 |
| 77 | Complex-II-Subunit | SDHB | V | 0.9825343 | 0.96326469 | 1.07906282 | 1.078375 | 0.9961674 |
| 78 | Connexin-43 | CNST43 | C | 1.04220251 | 0.90137086 | 0.75290307 | 1.02307123 | 0.9961674 |
| 79 | Coup-TFII | NR2F2 | C | 0.99942445 | 1.0575157 | 0.87414029 | 1 | 1.01866172 |
| 80 | Cox-IV | PTGS3 | C | 1.04013844 | 1.06415913 | 1.14569117 | 1 | 0.96870984 |
| 81 | Cox2 | CMC2 | C | 1 | 1.04476403 | 1.32006101 | 0.97865841 | 1.01806526 |
| 82 | CXCR4 | CXCR4 | C | 1 | 0.96172764 | 1.14159364 | 0.93182983 | 1 |
| 83 | Cyclin-B1 | CCNB1 | V | 1.07349257 | 1.05872657 | 0.94380768 | 1 | 0.99275254 |
| 84 | Cyclin-D1 | CCND1 | V | 0.96399748 | 1.03502904 | 1.12893794 | 1 | 1.02659112 |
| 85 | Cyclin-E1 | CCNE1 | V | 1.0014391 | 1.0203307 | 0.93578606 | 1 | 0.9814898 |
| 86 | Cyclophilin-F | PPIF | V | 1.05119742 | 0.92231509 | 1.1805286 | 0.95861048 | 0.9961674 |
| 87 | D-a-Tubulin | TUBA1A | V | 0.94375454 | 0.97172331 | 1.15525617 | 1 | 1.01661163 |
| 88 | DJ1 | PARK7 | V | 1 | 0.95260877 | 1.08023623 | 0.99387847 | 1.02420237 |
| 89 | DM-Histone-H3 | HISTH3 | V | 0.97334251 | 1.02099357 | 1.08550334 | 1 | 1.02278563 |
| 90 | DM-K9-Histone-H3 | H3K9ME2 | C | 0.99408992 | 1.0185009 | 0.99714629 | 1 | 0.99890451 |
| 91 | DUSP4 | DUSP4 | V | 1.18376353 | 1.05236299 | 0.86476433 | 0.99595526 | 0.997189 |
| 92 | Dvl3 | DVL3 | V | 0.97692368 | 0.93547195 | 1.01958141 | 1 | 1.02898546 |
| 93 | E-Cadherin | CDH1 | V | 0.87898958 | 0.78747224 | 0.96268167 | 1 | 1.02765104 |
| 94 | E2F1 | E2F1 | V | 1 | 1.03729852 | 0.97994941 | 0.99846202 | 1.04076562 |
| 95 | eEF2 | EEF2 | C | 1 | 0.95519031 | 2.14621811 | 0.96172676 | 1.28380933 |
| 96 | eEF2K | EEF2K | V | 0.98625904 | 0.80126985 | 1.14509445 | 1 | 1.07867977 |
| 97 | EGFR | EGFR | V | 0.92516225 | 0.89017515 | 0.86888006 | 1 | 1.07322009 |
| 98 | EGFR_pY1068 | EGFR | C | 4.40080168 | 1.19545149 | 0.86476433 | 0.8525339 | 0.85826054 |
| 99 | EGFR_pY1173 | EGFR | V | 2.11810671 | 1.06865188 | 0.97437496 | 0.98460361 | 0.9961674 |
| 100 | eIF4E | EIF4E | V | 1.02689446 | 0.98655728 | 1.02629989 | 1 | 0.97805541 |
| 101 | eIF4G | EIF4G1 | C | 1.00172537 | 0.97070565 | 0.86808637 | 1 | 0.99316201 |
| 102 | Elk1_pS383 | ELK1 | C | 0.99705695 | 1.05154311 | 1.08253536 | 1 | 1.00169205 |
| 103 | EMA | EMA | C | 1 | 1.02423411 | 0.82788633 | 1.00030419 | 1.01506313 |
| 104 | ER | ESR1 | V | 0.98714344 | 1.10987414 | 0.8374399 | 1.20314774 | 1.21680066 |
| 105 | ERCC1 | ERCC1 | V | 1 | 0.99666694 | 1.18265143 | 0.94490032 | 1.00632382 |
| 106 | ERCC5 | ERCC5 | C | 0.90348937 | 0.93433643 | 1.35429094 | 1 | 1.22748855 |
| 107 | Ets-1 | ETS1 | V | 1 | 0.99835625 | 1.80275091 | 0.97180137 | 1.012855 |
| 108 | FAK | PTK2 | C | 0.93675578 | 0.88059939 | 1.38489281 | 1 | 1.11957917 |
| 109 | FAK_pY397 | PTK2 | V | 1.0771272 | 1.10987414 | 0.83167525 | 1.06853751 | 0.92382188 |
| 110 | FASN | FASN | V | 0.97062326 | 0.94471387 | 0.86476433 | 1.00588518 | 0.99793396 |
| 111 | Fibronectin | FN1 | V | 1 | 0.9805865 | 0.97741873 | 1.00221497 | 0.98839258 |
| 112 | FoxM1 | FOXM1 | V | 0.99669549 | 1.0306824 | 0.95109016 | 1.03763244 | 0.9961674 |
| 113 | FoxO3a | FOX3 | C | 1 | 1.0786417 | 0.92889173 | 1.02731511 | 0.98695952 |
| 114 | FoxO3a_pS318_S321 | FOXO3 | C | 1 | 1.04954422 | 1.19889868 | 0.99075177 | 1.10835899 |
| 115 | FRA-1 | FOSL1 | C | 1.19757534 | 0.986861 | 1.12745488 | 0.97673382 | 0.9961674 |
| 116 | G6PD | G6PD | V | 1 | 0.87770409 | 1.2529094 | 0.87524329 | 1.03557536 |
| 117 | Gab2 | GAB2 | V | 1.00752478 | 0.92818537 | 0.94714538 | 0.95341621 | 0.9961674 |
| 118 | GAPDH | GAPDH | C | 1.00403937 | 0.95755662 | 0.72388791 | 1.04381146 | 0.9961674 |
| 119 | GATA3 | GATA3 | V | 1 | 1.00720651 | 1.18077695 | 0.98387473 | 1.00069562 |
| 120 | GCN5L2 | KAT2A | V | 1.01230467 | 1.0395337 | 0.86717609 | 1 | 0.98945945 |
| 121 | Glutamate-D1-2 | GLUD | C | 1 | 1.02353622 | 1.10777462 | 0.9771624 | 1.07814458 |
| 122 | Glutaminase | GLS | C | 0.95400596 | 0.97863606 | 1.13393689 | 1 | 1.00462713 |
| 123 | GPBB | PYGM | V | 1 | 0.94767497 | 1.29604455 | 0.95235915 | 1.08065471 |
| 124 | GSK-3a-b | GSK3A/GSK3B | V | 1.01771979 | 1.04268839 | 1.1819356 | 0.95891159 | 0.9961674 |
| 125 | GSK-3a-b_pS21_S9 | GSK3A GSK3B | V | 1.07007471 | 1.08083735 | 0.95573748 | 1 | 0.9136389 |
| 126 | Gys | GYS1 | V | 1.02811028 | 0.94143347 | 0.86476433 | 1.09811508 | 0.8748025 |
| 127 | Gys_pS641 | GYS1 | V | 0.80667052 | 0.82829438 | 1.06230235 | 1 | 1.00541579 |
| 128 | H2AX_pS140 | H2AFX | C | 0.97235354 | 0.96219959 | 1.16070306 | 1 | 1.11173595 |
| 129 | HER2 | ERBB2 | V | 1.07213224 | 0.99170562 | 1.08050809 | 0.90991804 | 0.9961674 |
| 130 | HER2_pY1248 | ERBB2 | C | 2.74627437 | 1.10987414 | 0.94241107 | 0.99152638 | 0.98035874 |
| 131 | HER3 | ERBB3 | V | 0.99522936 | 1.04864248 | 0.91068033 | 1.01469683 | 0.9961674 |
| 132 | HER3_pY1289 | ERBB3 | C | 1 | 0.90135378 | 1.3524923 | 0.97023048 | 1.05074746 |
| 133 | Heregulin | NRG1 | V | 0.98350624 | 0.98500523 | 1.01927515 | 1 | 1.03004257 |
| 134 | HES1 | HES1 | V | 1.255921 | 1.09851326 | 0.91669123 | 0.99287935 | 0.9961674 |
| 135 | Hexokinase-II | HK2 | V | 0.96246186 | 1.00508383 | 0.99068221 | 1.00571449 | 0.9961674 |
| 136 | HIAP | BIRC2 | C | 0.9805298 | 0.98019257 | 0.92790207 | 1.01243683 | 0.9961674 |
| 137 | Hif-1-alpha | HIF1A | C | 1.01018177 | 1.05845283 | 0.83402319 | 1.01670268 | 0.9961674 |
| 138 | Histone-H3 | HIST3H3 | V | 0.96565174 | 0.99705015 | 1.30918019 | 1 | 1.06794811 |
| 139 | HSP27 | HSBP1 | C | 1 | 1.05301238 | 1.12071401 | 0.99072141 | 1.040921 |
| 140 | HSP27_pS82 | HSBP1 | V | 1.02971497 | 0.94954448 | 0.86200009 | 1 | 1.05902467 |
| 141 | HSP70 | HSPA1A | C | 0.97861321 | 0.90903761 | 1.19746441 | 1 | 1.00730039 |
| 142 | IGF1R_pY1135_Y1136 | IGF1R | V | 0.99455151 | 1.07980426 | 0.9300401 | 1 | 0.99782616 |
| 143 | IGFBP2 | IGFBP2 | V | 1 | 0.89756044 | 0.81056983 | 1.34625926 | 1.32061521 |
| 144 | IGFBP5 | IGFBP5 | C | 1.02838713 | 1.05953367 | 0.85942523 | 1 | 1.04027714 |
| 145 | IGFRb | IGF1R | C | 0.98990339 | 1.00794896 | 1.29408458 | 1 | 1.02646227 |
| 146 | INPP4b | INPP4B | V | 0.97091417 | 1.01714464 | 1.11545111 | 1 | 1.00717301 |
| 147 | IRF-1 | IRF1 | C | 1.00709292 | 0.99345725 | 1.20854246 | 0.9342585 | 0.9961674 |
| 148 | IRS1 | IRS1 | V | 0.99973413 | 1.03578262 | 0.98703599 | 1 | 1.01579225 |
| 149 | JAB1 | JAB1 | C | 1 | 1.04546663 | 1.0310956 | 0.93969967 | 1.02366361 |
| 150 | Jagged1 | JAG1 | V | 1.0300136 | 1.02964541 | 0.85886051 | 1.02285714 | 0.9961674 |
| 151 | Jak2 | JAK2 | V | 0.98549408 | 1.01143288 | 1.07774582 | 1 | 1.05042913 |
| 152 | JNK2 | MAPK9 | C | 0.89470009 | 0.95778941 | 1.67568564 | 1 | 1.22391787 |
| 153 | JNK_pT183_Y185 | MAPK8 | V | 0.93398482 | 0.98897415 | 1.04880451 | 1 | 1.01592683 |
| 154 | LC3A-B | LC3AB | C | 1.12154513 | 1.00818528 | 1.22732348 | 0.90513179 | 0.9961674 |
| 155 | Lck | LCK | V | 0.92254821 | 0.98600017 | 1.50056409 | 1 | 1.06699788 |
| 156 | LDHA | LDHA | C | 0.95092862 | 1.01497625 | 1.71366833 | 1 | 1.11236765 |
| 157 | MAPK_pT202_Y204 | MAPK3 | V | 1 | 1.05799357 | 0.9923906 | 1.00215867 | 0.98325467 |
| 158 | Mcl-1 | MCL1 | V | 1.20401446 | 1.04760455 | 0.92838488 | 0.99927157 | 0.9961674 |
| 159 | MCT4 | SLC16A4 | V | 1 | 1.0938093 | 1.32707011 | 1.00952142 | 0.99523463 |
| 160 | MDM2_pS166 | MDM2 | V | 1.17537457 | 1.08417422 | 1.22122437 | 0.9769832 | 0.9961674 |
| 161 | MEK1 | MAP2K1 | V | 1 | 0.95330597 | 1.15101453 | 0.99595295 | 1.01369526 |
| 162 | MEK1_pS217_S221 | MAP2K1 | V | 1.00507944 | 0.98660479 | 1.06707528 | 0.96196219 | 0.9961674 |
| 163 | MEK2 | MAP2K2 | V | 0.95233477 | 0.96544984 | 1.03995455 | 1 | 1.00265576 |
| 164 | Merlin | NF2 | C | 0.96707616 | 0.84611332 | 1.29919517 | 1 | 1.10608659 |
| 165 | MIF | MIF | C | 1.05930034 | 1.07570908 | 0.92980609 | 1 | 0.95178017 |
| 166 | MIG6 | ERRFI1 | V | 1.03506518 | 1.03016327 | 1.2385752 | 0.97962475 | 0.9961674 |
| 167 | Mitochondria | MTCO2 | V | 1.00941447 | 0.9566033 | 0.7987916 | 1.0296128 | 0.9961674 |
| 168 | MMP2 | MMP2 | V | 0.98452595 | 1.0179352 | 1.01929223 | 1 | 1.03393572 |
| 169 | Mnk1 | MKNK1 | V | 1.00595283 | 1.01694021 | 0.88238643 | 1 | 0.98833624 |
| 170 | MSH2 | MSH2 | V | 1 | 1.07746321 | 0.69615847 | 1.13246589 | 0.99996993 |
| 171 | MSH6 | MSH6 | C | 1 | 0.91837324 | 1.14264673 | 0.95166016 | 1.03963466 |
| 172 | mTOR | MTOR | V | 1 | 0.96808961 | 0.93492683 | 0.99259338 | 1.00534756 |
| 173 | mTOR_pS2448 | MTOR | C | 1.15053434 | 0.94731662 | 1.46520466 | 0.92120448 | 0.9961674 |
| 174 | Myosin-11 | MYH11 | V | 0.939312 | 1.00623186 | 0.98914097 | 1.03526585 | 0.9961674 |
| 175 | Myosin-IIa_pS1943 | MYO2A | V | 1.02901782 | 1.01545003 | 1.35970462 | 1 | 0.99597494 |
| 176 | Myt1 | MYT1 | C | 0.95182403 | 1.05167365 | 0.89523578 | 1 | 1.03538617 |
| 177 | N-Cadherin | CDH2 | V | 0.94238029 | 1.01109687 | 1.14527786 | 1 | 1.05699329 |
| 178 | N-Ras | NRAS | V | 1.00680855 | 1.04657302 | 0.86476433 | 0.98654795 | 1.00043406 |
| 179 | NAPSIN-A | NAPSA | C | 0.94374539 | 1.01402751 | 1.03793425 | 1 | 1.00680983 |
| 180 | NDRG1_pT346 | NDRG1 | V | 0.98687438 | 0.98400058 | 0.87047775 | 1.0314944 | 0.9961674 |
| 181 | NDUFB4 | NDUFB4 | V | 1 | 1.06308378 | 0.87537915 | 1.01901342 | 0.99505667 |
| 182 | NF-kB-p65_pS536 | NFKB1 | C | 1.01339467 | 0.89853326 | 1.12842013 | 0.97440968 | 0.9961674 |
| 183 | Notch1 | NOTCH1 | V | 0.95468382 | 0.89218482 | 0.895374 | 1.01629225 | 0.9961674 |
| 184 | Notch3 | NOTCH3 | C | 0.99661129 | 1.00271174 | 0.90606515 | 1.01040795 | 0.9961674 |
| 185 | P-Cadherin | CDH3 | C | 0.95251942 | 1 | 1.07151744 | 1.01316341 | 0.9961674 |
| 186 | p16INK4a | CDKN2A | V | 1 | 1.07774596 | 0.77033989 | 1.0941682 | 1.00176386 |
| 187 | p21 | CDKN1A | V | 0.86634721 | 0.94474534 | 1.15893299 | 1 | 1.08475554 |
| 188 | p27-Kip-1 | CDKN1B | V | 0.9615962 | 1.00430311 | 0.90160083 | 1.01042496 | 0.9961674 |
| 189 | p27_pT157 | CDKN1B | C | 0.99309374 | 1.0092162 | 1.21752943 | 1 | 1.07265336 |
| 190 | p27_pT198 | CDKN1B | V | 0.98944366 | 0.99205549 | 0.9424632 | 1.01407088 | 0.9961674 |
| 191 | p38-MAPK | MAPK14 | V | 0.98985327 | 0.94978541 | 0.93730754 | 1 | 0.99797747 |
| 192 | p38_pT180_Y182 | MAPK14 | V | 1.05927792 | 1.0100974 | 0.97067149 | 1 | 0.95441386 |
| 193 | p44-42-MAPK | MAPK3 | V | 0.97588656 | 0.82403224 | 0.94232214 | 1.01641364 | 0.9961674 |
| 194 | p53 | TP53 | C | 1 | 0.98358388 | 1.27572412 | 0.92544348 | 1.06109454 |
| 195 | p70-S6K1 | RPS6KB1 | V | 0.97114758 | 0.97297844 | 0.92840595 | 1.04544119 | 0.9961674 |
| 196 | p70-S6K_pT389 | RPS6KB1 | V | 1.2935726 | 1.04461118 | 1.09688684 | 0.96753818 | 0.9961674 |
| 197 | p90RSK_pT573 | RPS6K | C | 1.06810585 | 1.04696678 | 0.96728924 | 0.98735146 | 0.9961674 |
| 198 | PAI-1 | SERPINE1 | V | 1.64278432 | 0.85267672 | 0.86476433 | 0.97943254 | 1.11029252 |
| 199 | PAR | PAR | C | 1.02831817 | 0.88765835 | 0.86476433 | 0.91785084 | 1.02415556 |
| 200 | PARP1 | PARP1 | V | 0.97679387 | 0.97434699 | 0.86476433 | 1.19220007 | 1.02760106 |
| 201 | Paxillin | PXN | C | 1 | 0.91393183 | 1.23493058 | 0.93259152 | 1.1237741 |
| 202 | PCNA | PCNA | C | 1.04512967 | 1.01280078 | 1.54518571 | 0.92017765 | 0.9961674 |
| 203 | PD-L1 | CD274 | C | 1.03487459 | 1.04758511 | 0.94371806 | 1 | 0.97554535 |
| 204 | Pdcd-1L1 | PDCD1 | C | 1.010816 | 1.0467595 | 0.97828505 | 0.94992465 | 0.9961674 |
| 205 | Pdcd4 | PDCD4 | C | 1.1177794 | 1.06934018 | 0.89510559 | 1 | 0.96845108 |
| 206 | PDGFR-b | PDGFR | V | 0.86110083 | 0.90779296 | 0.90302781 | 1 | 1.02625315 |
| 207 | PDK1 | PDK1 | V | 0.93024977 | 0.9485304 | 1.07265868 | 1 | 1.0061328 |
| 208 | PDK1_pS241 | PDK1 | V | 1 | 0.8523808 | 0.88611307 | 1.01304293 | 0.9586902 |
| 209 | PEA-15 | PEA15 | V | 0.9894607 | 0.89169858 | 0.92465195 | 1 | 1.00907529 |
| 210 | PEA-15_pS116 | PEA15 | V | 1 | 1.0473262 | 1.01837085 | 0.95148628 | 1.06496621 |
| 211 | PI3K-p110-a | PIK3C2A | C | 1 | 1.02939303 | 0.8809253 | 0.99658394 | 1.03004403 |
| 212 | PI3K-p110-b | PIK3BC | C | 1 | 1.0436906 | 0.91272706 | 0.97119549 | 1.00933552 |
| 213 | PI3K-p85 | PIK3R1 | V | 0.96764657 | 0.99657139 | 0.8969759 | 1.00725295 | 0.9961674 |
| 214 | PKA-a | PRKAR1A | V | 1.07678249 | 0.9500368 | 0.88910923 | 1 | 0.92718611 |
| 215 | PKC-a | PRKCA | V | 1.09148967 | 0.9671185 | 1.14049611 | 0.96127103 | 0.9961674 |
| 216 | PKC-a_pS657 | PRKCA | C | 1 | 0.9864159 | 1.16561912 | 0.98372456 | 1.05305489 |
| 217 | PKC-b-II_pS660 | PRKCB | V | 1.07585781 | 1.04856202 | 0.90115396 | 1 | 0.97240447 |
| 218 | PKC-delta_pS664 | PRKCD | V | 0.98722775 | 1.04694087 | 0.88747027 | 1.04049419 | 0.9961674 |
| 219 | PKM2 | PKM2 | C | 0.99683104 | 0.83147748 | 1.22299524 | 1 | 1.39213741 |
| 220 | PLC-gamma2_pY759 | PLCG2 | C | 1.02322096 | 1.00573332 | 1.08151753 | 0.9555969 | 0.9961674 |
| 221 | PLK1 | PLK1 | C | 1.09292059 | 1.0050283 | 0.82414986 | 1.04807539 | 0.9961674 |
| 222 | PMS2 | PMS2 | V | 1 | 0.9800512 | 1 | 0.9890026 | 1.02514036 |
| 223 | Porin | VDAC1 | V | 0.99036194 | 1.01560259 | 1.0847959 | 1 | 1.00737068 |
| 224 | PR | PGR | V | 1 | 1.00015609 | 1.15726317 | 0.99673156 | 0.99627897 |
| 225 | PRAS40 | AKT1S1 | C | 0.96076258 | 0.93416342 | 0.86476433 | 1.06161781 | 1.03878245 |
| 226 | PRAS40_pT246 | AKT1S1 | V | 1.15797066 | 1.0518479 | 0.91262334 | 1 | 0.99379031 |
| 227 | PREX1 | PREX1 | V | 0.89532322 | 0.96645241 | 0.87051607 | 1 | 1.0072565 |
| 228 | PTEN | PTEN | V | 0.89557521 | 0.87887864 | 1.11201956 | 1 | 1.05142426 |
| 229 | Puma | BBC3 | C | 1.01446618 | 1.03574447 | 1.07695543 | 0.94059562 | 0.9961674 |
| 230 | PYGM | PYGM | C | 1.03333816 | 1.0577226 | 1.38986653 | 0.97495108 | 0.9961674 |
| 231 | Rab11 | RAB11A | E | 1 | 0.96338705 | 1.1503278 | 0.95277055 | 1.04241169 |
| 232 | Rab25 | RAB25 | V | 1 | 1.00562535 | 0.89242038 | 0.98237485 | 1.00208539 |
| 233 | Rad50 | RAD50 | V | 0.9494644 | 0.97847884 | 1.17789068 | 1 | 1.07439597 |
| 234 | Rad51 | RAD51 | V | 1.01194779 | 1.00408148 | 0.92697255 | 0.95492724 | 0.9961674 |
| 235 | Raptor | RPTOR | V | 0.96004276 | 0.94006881 | 1.19048868 | 1 | 1.03452995 |
| 236 | Rb | RB1 | Q | 1.09088738 | 1.08699791 | 1.25347472 | 1 | 0.98003257 |
| 237 | RBM15 | RBM15 | V | 1 | 0.93621415 | 1.09394655 | 0.98187221 | 1.04386325 |
| 238 | Rb_pS807_S811 | RB1 | V | 1.20552561 | 1.07127846 | 1.11299569 | 0.99473509 | 0.9961674 |
| 239 | Rheb | RHEB | C | 1.00713125 | 1.05002493 | 1.36075801 | 0.96931122 | 0.9961674 |
| 240 | Rictor | RICTOR | C | 0.99902626 | 1.01601024 | 0.8817106 | 1.01581517 | 0.9961674 |
| 241 | Rictor_pT1135 | RICTOR | V | 1.04597237 | 1.01933948 | 1.10753293 | 0.97327936 | 0.9961674 |
| 242 | Rock-1 | ROCK1 | C | 1.0968927 | 1.10987414 | 1.41604047 | 0.95350453 | 0.95569427 |
| 243 | RPA32 | RPA32 | C | 1.00805412 | 0.98726826 | 1.28197203 | 0.89391722 | 0.9961674 |
| 244 | RPA32_pS4_S8 | RPA32 | C | 1.12032556 | 1.03601747 | 1.14493817 | 1 | 0.95515584 |
| 245 | RSK | RPS6KA1 | C | 0.92248513 | 0.98882584 | 0.96038459 | 1 | 1.00815691 |
| 246 | S6 | RPS6 | V | 1 | 1.13100339 | 0.68942367 | 1.14529533 | 0.90801569 |
| 247 | S6_pS235_S236 | RPS6 | V | 4.51469106 | 1.71664189 | 0.86476433 | 0.79556729 | 0.75772632 |
| 248 | S6_pS240_S244 | RPS6 | V | 2.13631071 | 1.10987414 | 0.89373461 | 0.60004853 | 0.61064364 |
| 249 | SCD | SCD | V | 1 | 0.97362264 | 0.96314248 | 0.98033868 | 1.02657597 |
| 250 | SDHA | SDHA | V | 0.9368612 | 1.00804053 | 0.96671862 | 1 | 1.03295355 |
| 251 | SF2 | SRSF1 | V | 0.93971627 | 0.95626891 | 1.04728663 | 1.01044883 | 0.9961674 |
| 252 | Shc_pY317 | SHC1 | V | 1.40869046 | 1.01218322 | 1.02248645 | 0.96687515 | 0.9961674 |
| 253 | SHP-2_pY542 | PTPN11 | C | 1.76739451 | 0.99580614 | 0.95521505 | 0.94983641 | 0.9961674 |
| 254 | SLC1A5 | SLC1A5 | C | 1.03723535 | 0.91387793 | 0.94680203 | 0.89715204 | 0.9961674 |
| 255 | Smac | DIABLO | Q | 0.93049795 | 0.94870552 | 1.10296679 | 1 | 1.07410709 |
| 256 | Smad1 | SMAD1 | V | 0.97752645 | 0.97908973 | 1.09301357 | 1 | 1.08181871 |
| 257 | Smad3 | SMAD3 | V | 1.01588268 | 0.97525167 | 1.00659759 | 0.98163666 | 0.9961674 |
| 258 | Smad4 | SMAD4 | V | 0.87606815 | 1.02810294 | 0.94740143 | 1.0036719 | 0.9961674 |
| 259 | Snail | SNAI2 | Q | 1.0166748 | 1.06355106 | 0.8842325 | 1 | 0.97804048 |
| 260 | SOD2 | SOD2 | V | 0.92356816 | 0.88992671 | 0.86680234 | 1 | 1.04463858 |
| 261 | Sox2 | SOX2 | V | 1.01567556 | 1.05149078 | 1.01533331 | 1 | 0.9780345 |
| 262 | Src | SRC | V | 1 | 1.04183723 | 0.80575852 | 1.12974352 | 1.04386616 |
| 263 | Src_pY416 | SRC | V | 1.34406784 | 0.97928267 | 0.89721061 | 1 | 0.96466483 |
| 264 | Src_pY527 | SRC | V | 1 | 0.81067831 | 0.95378191 | 0.93052178 | 1.01972326 |
| 265 | Stat3 | STAT3 | C | 0.89636499 | 0.81051231 | 1.11805902 | 1 | 1.03391349 |
| 266 | Stat3_pY705 | STAT3 | V | 1.10165195 | 0.88164876 | 1.07424418 | 0.95821435 | 0.9961674 |
| 267 | Stat5a | STAT5A | V | 0.98795721 | 1.04424233 | 1.09096096 | 1.0077482 | 0.9961674 |
| 268 | Stathmin-1 | STMN1 | V | 0.99553381 | 1.03288995 | 1.00330812 | 1 | 1.01150665 |
| 269 | Syk | SYK | V | 1 | 1.04739185 | 0.83056756 | 1.09095515 | 1.15559786 |
| 270 | Tau | MAPT | C | 0.91662586 | 0.97239986 | 1.2486163 | 1 | 1.10387715 |
| 271 | TAZ | TAZ | V | 1.23851829 | 0.96456155 | 0.92153104 | 1 | 0.94436225 |
| 272 | TFAM | TFAM | V | 0.97339249 | 1.03244757 | 0.91568493 | 1 | 1.07099059 |
| 273 | TFRC | TFRC | V | 1 | 1.01633781 | 1.40633083 | 0.98283878 | 1.03169585 |
| 274 | TIGAR | TIGAR | V | 0.99152474 | 1.00519695 | 1.35998779 | 1 | 1.10275526 |
| 275 | Transglutaminase | TGM2 | V | 0.95952039 | 1.01503277 | 0.95313632 | 1.02162288 | 0.9961674 |
| 276 | TSC1 | TSC1 | C | 1.01081806 | 0.94300593 | 0.925499 | 1 | 0.97717241 |
| 277 | TTF1 | TTF1 | V | 1 | 0.99923795 | 1.51299904 | 0.97093894 | 1.05661407 |
| 278 | Tuberin | TSC2 | V | 1.01218845 | 0.95853597 | 0.96351418 | 1 | 0.98200092 |
| 279 | Tuberin_pT1462 | TSC2 | V | 1 | 1.04709907 | 1.33067437 | 0.99664872 | 1.03975199 |
| 280 | TWIST | TWIST1 | C | 0.96503772 | 1.0540671 | 0.88361757 | 1.02981268 | 0.9961674 |
| 281 | Tyro3 | TYRO3 | V | 0.92101674 | 0.97756234 | 1.05524096 | 1 | 1.03486423 |
| 282 | UBAC1 | UBAC1 | V | 1.07611115 | 1.05065688 | 1.03111712 | 1 | 0.96319562 |
| 283 | Ubq-Histone-H2B | H2BFM | C | 1 | 1.00639247 | 0.86921699 | 0.94137635 | 1.00826983 |
| 284 | UGT1A | UGT1A | V | 0.99356075 | 1.00571414 | 1.13631134 | 1 | 1.12200563 |
| 285 | VEGFR-2 | KDR | V | 1 | 0.90340707 | 1.0298491 | 0.97628903 | 1.04701945 |
| 286 | VHL-EPPK1 | VHL | E | 1 | 0.86036418 | 1.13637723 | 1.01110956 | 0.91973947 |
| 287 | Vimentin | VIM | C | 0.98262134 | 1.14544206 | 1.11638209 | 1 | 0.98805708 |
| 288 | Wee1 | WEE1 | C | 1 | 1.04098951 | 0.92287229 | 0.96562771 | 1.0185584 |
| 289 | XBP-1 | XBP1 | C | 1.00727539 | 0.98635859 | 0.99835781 | 0.9042901 | 0.9961674 |
| 290 | XIAP | XIAP | C | 0.99545301 | 0.97230523 | 1.0563857 | 1 | 1.04588419 |
| 291 | XPA | XPA | V | 1.06136008 | 1.06112921 | 0.84384942 | 1 | 1.0283375 |
| 292 | XPF | XPF | C | 1 | 1.08593536 | 0.81690996 | 1.07337598 | 1.01494623 |
| 293 | XRCC1 | XRCC1 | C | 0.94964566 | 0.98888585 | 1.11749467 | 1 | 1.03312641 |
| 294 | YAP | YAP1 | E | 1 | 1.02825031 | 0.92499098 | 1.02014594 | 0.98916712 |
| 295 | YAP_pS127 | YAP1 | E | 1.03706221 | 0.9746777 | 0.86476433 | 1.02543596 | 0.99078023 |
| 296 | YB1 | YBX1 | V | 1.00239049 | 0.96200454 | 1.12775845 | 0.97214401 | 0.9961674 |
| 297 | YB1_pS102 | YBX1 | V | 1.02158252 | 0.92466686 | 1.02494173 | 0.92513189 | 0.9961674 |
| 298 |  |  |  |  |  |  |  |  |
| 299 |  |  | CF | 1 | 0.90100306 | 1.15638443 | 1 | 1.00384734 |
